# Sex differences in kidney gene expression during the life cycle of F344 rats

**DOI:** 10.1186/2042-6410-4-14

**Published:** 2013-07-31

**Authors:** Joshua C Kwekel, Varsha G Desai, Carrie L Moland, Vikrant Vijay, James C Fuscoe

**Affiliations:** 1 Personalized Medicine Branch, Division of Systems Biology, National Center for Toxicological Research, US Food and Drug Administration, 3900 NCTR Road, Jefferson, AR 72079, USA

**Keywords:** Kidney, Gene expression, Sex, Age, Biomarker

## Abstract

**Background:**

The kidney functions in key physiological processes to filter blood and regulate blood pressure via key molecular transporters and ion channels. Sex-specific differences have been observed in renal disease incidence and progression, as well as acute kidney injury in response to certain drugs. Although advances have been made in characterizing the molecular components involved in various kidney functions, the molecular mechanisms responsible for sex differences are not well understood. We hypothesized that the basal expression levels of genes involved in various kidney functions throughout the life cycle will influence sex-specific susceptibilities to adverse renal events.

**Methods:**

Whole genome microarray gene expression analysis was performed on kidney samples collected from untreated male and female Fischer 344 (F344) rats at eight age groups between 2 and 104 weeks of age.

**Results:**

A combined filtering approach using statistical (ANOVA or pairwise *t* test, FDR 0.05) and fold-change criteria (>1.5 relative fold change) was used to identify 7,447 unique differentially expressed genes (DEGs). Principal component analysis (PCA) of the 7,447 DEGs revealed sex-related differences in mRNA expression at early (2 weeks), middle (8, 15, and 21 weeks), and late (104 weeks) ages in the rat life cycle. Functional analysis (Ingenuity Pathway Analysis) of these sex-different genes indicated over-representation of specific pathways and networks including renal tubule injury, drug metabolism, and immune cell and inflammatory responses. The mRNAs that code for the qualified urinary protein kidney biomarkers KIM-1, Clu, Tff3, and Lcn2 were also observed to show sex differences.

**Conclusions:**

These data represent one of the most comprehensive in-life time course studies to be published, assessing sex differences in global gene expression in the F344 rat kidney. PCA and Venn analyses reveal specific periods of sexually dimorphic gene expression which are associated with functional categories (xenobiotic metabolism and immune cell and inflammatory responses) of key relevance to acute kidney injury and chronic kidney disease, which may underlie sex-specific susceptibility. Analysis of the basal gene expression patterns of renal genes throughout the life cycle of the rat will improve the use of current and future renal biomarkers and inform our assessments of kidney injury and disease.

## Background

The kidney functions in the fundamental physiological processes of filtering waste in the blood and re-absorbing water and small molecules through specific molecular transporters. Both endogenous and exogenous metabolites, hormones, and other molecules are handled primarily by proteins in the ATP-binding cassette and solute carrier super-families. A variety of organic substrates including peptides, nucleosides, drugs, and food constituents are recognized and transported by these proteins [1,2]. Homeostatic control of blood pressure is another key activity of the kidney via sodium-hydrogen ion exchangers that are regulated by the renin-angiotensin system [3].

Sex differences have been well established for various renal diseases, with females generally having protection compared to males through estrogen’s inhibitory, but androgen’s activating, behaviors on the renin-angiotensin system [4]. Females generally show lower risk for chronic kidney disease [5-8] compared to males. However, not all renal diseases are less prevalent in females; higher risk in females for diabetic kidney disease has been reported [9]. Diabetic vs. non-diabetic nephropathies present with conflicting reports from the literature as to their sex-specificity of injury [3].

Additionally, sex-related differences in acute kidney injury and nephrotoxicity are also well established, although heterogeneous in their sex specificity. Many reports suggest that females show susceptibility to drug-, diet-, and food supplement-induced renal injury [10-12]. However, male experimental animals have been shown to be more susceptible to certain metal-induced nephrotoxicity than females [13]. Females also show protection against obesity-related adverse renal events due to decreased inflammatory response [14] compared to males.

The molecular mechanisms responsible for these sex differences in adverse events are not well understood. However, some sex-specific toxicities have been shown to be related to expression level differences of specific proteins and transporters. For example, female rats exhibit sex-specific protection against mercury-induced nephrotoxicity compared to males [13]. This protection is conferred to females via the lower expression of organic anion transporters 1 and 3 (Oat1, Oat3) in the proximal tubules compared to males, limiting the uptake of mercury into renal cells and preventing nephrotoxicity. The sex-specific expression difference of various proteins and transporters may be responsible for other sex-related differences in renal disease and injury as well. Therefore, a better understanding of the basal expression levels in both male and female animals, across the entire rat life cycle will provide valuable data for assessing sex differences in the kidney.

Drug-induced kidney injury is a common reason for drug candidate failure during drug development [15]. Apart from histopathological evaluations, classical markers of kidney injury via blood (blood urea nitrogen, creatinine) and urinary endpoints (color, specific gravity, and protein levels) are generally considered to be insensitive and non-specific for detecting kidney injury [16]. Collaborative efforts between pharmaceutical and regulatory groups and the Predictive Safety Testing Consortium have led to the evaluation and qualification of several urinary protein biomarkers that serve as preclinical diagnostic biomarkers of drug-induced kidney injury including KIM-1, clusterin, trefoil factor 3, and osteopontin [17]. These new biomarkers are altered before major functional defects have occurred and signal specific renal damage (KIM-1, Clu). Additional urinary protein biomarkers, genes, and metabolites are being similarly evaluated for inclusion as preclinical biomarkers of toxicity [18]. However, most of the in-life testing and evaluation of renal toxicity and biomarker performance occur exclusively in male animals. Thus, further investigation into female and sex-related differences in urinary protein biomarkers specifically expressed in the kidney is warranted.

Knowledge of the basal expression of these genes and their normal, sex-dependent differences throughout the life cycle in preclinical models will inform the evaluation of these biomarkers and deepen our understanding of kidney function. We report global gene expression profiles of the kidney across the rat life cycle and the sex-specific changes that may influence normal kidney biology or underlie susceptibilities to adverse events.

## Methods

### Animal study

Male and female Fischer 344 (F344) rats were obtained and housed as previously described [19] and housed under AAALAC-approved conditions with a 12-h light/dark cycle (0600–1800). The rats were housed two per cage in standard polycarbonate cages with hardwood chip bedding maintained at 23°C with a relative humidity of approximately 50%. The rats were sacrificed at 2, 5, 6, 8, 15, 21 weeks (*n* = 6 per age group), 78 weeks (*n* = 8), and 104 weeks (*n* = 6) of age. Females were not synchronized for estrous cycle. Fischer rats were used due to their frequent use for in-life bioassays in preclinical toxicology studies. The rats were treated according to the National Center for Toxicological Research Institutional Animal Care and Use Committee guidelines.

### Necropsy

The animals were sacrificed at the same circadian time for each time point and euthanized through carbon dioxide asphyxiation. The sections of the kidneys were collected from rats aged 78 and 104 weeks for histological examination and placed in 10% neutral buffered formalin. Standa paraffin embedding, sectioning, and hematoxylin and eosin staining were performed for histopathological examination of the 78- and 104-week kidney sections by a staff board-certified pathologist.

### RNA isolation

Total RNA was isolated from approximately 30 mg of whole, homogenized, kidney tissue of independent animals as previously described [19] using Qiagen RNeasy Mini Kit (Qiagen Inc., Valencia, CA, USA) according to manufacturer’s protocol. The yield of the extracted RNA was determined spectrophotometrically by measuring the optical density at 260 nm (Nanodrop-1000, Thermo Scientific, Wilmington, DE, USA). The purity and quality of extracted RNA were evaluated using the RNA 6000 LabChip and Agilent 2100 Bioanalyzer (Agilent Technologies, Palo Alto, CA, USA). The RNA samples with RNA integrity numbers (RINs) greater than 8.0 were used for microarray experiments with an average RIN of 8.5 for all samples.

### Microarray experiments

Whole genome microarray experiments (*n* = 4 for 2, 5, 6, 8, and 104-week males; *n* = 5 for 15, 21, and 78-week males and 2, 5, 6, 8, 15, 21, 78, and 104 week females) were completed for a total of 75 microarrays. Single color (Cy3) Agilent Whole Rat Genome 4 × 44 K microarrays and reagents were used according to manufacturer’s protocols (Agilent Technologies, Santa Clara, CA, USA) using 500 ng of total RNA. An Agilent one-color spike-in kit was used as a positive control. The arrays were scanned using the Agilent High Resolution C Scanner (Agilent Technologies). The images were analyzed using Agilent’s Feature Extraction software. A universal rat reference (URR) RNA (Stratagene, Agilent Technologies) was labeled and incorporated into the array design to control for batch/day effects during data processing. Pair-wise Pearson’s correlations between un-normalized individual URR array intensity values (F532-median) ranged from *R* = 0.955 to 0.991. Full microarray data are available at Gene Expression Omnibus with accession GSE47070.

### Microarray data analysis

Microarray intensity data were uploaded to the ArrayTrack™ database [20] and normalized using 75th-percentile scaling without background subtraction. Relative fold-change values (individual values divided by the averaged expression from all ages in both sexes) were calculated on a per spot/feature basis. Following the MAQC Consortium recommendations (MAQC Consortium, 2006) for microarray data analysis, we employed a combined approach using both fold change and statistical criteria to determine differential gene expression. Intensity values were log(2)-transformed prior to statistical analysis. Filtering criteria, consisting of a 1.5-fold change and ANOVA (FDR 5%) for age-related changes or 1.5-fold change and pairwise *t* test (FDR 5%) for sex-related changes, were used to define an initial set of differentially expressed genes (DEGs). Applying these criteria to the 43,379 features on the Agilent microarrays resulted in 841 unique genes showing sex-related differences and 7,274 unique genes showing age-related differences for a total of 7,447 differentially expressed genes. The complete dataset with annotations, fold changes, and statistical values is available in Additional files 1 and 2: Table SA1 and Table SA2. For brevity and consistency, the genes are referenced by their official gene symbol as defined by National Center for Biotechnology Information (NCBI). Three-dimensional principal component analysis (PCA) was performed on normalized intensity values of the 7,447 differentially expressed features in ArrayTrack (http://www.fda.gov/ScienceResearch/BioinformaticsTools/Arraytrack/default.htm). Default settings in the Ingenuity Pathway Analysis (IPA, http://www.ingenuity.com/) for expression dataset analyses were used for gene list functional analysis. Gene lists were uploaded using NCBI Entrez gene IDs or gene symbols and submitted for IPA Core Analysis. Ranked results from Top Networks, Bio-Functions, Tox-Functions, and Canonical Pathways meeting minimal *p* value <0.05 for each pathway containing at least three focus molecules were queried for functional annotations and over-represented pathways to facilitate the biological interpretation of selected gene lists.

### Microarray probe annotation update

The Agilent rat 4 × 44 K whole genome microarray contains a total of 45,220 probes per array, 1,841 of which are Agilent control probes. Therefore, 43,379 probes were updated for the most current annotation. The 07Feb2007 version of Agilent annotation file (http://www.chem.agilent.com/cag/bsp/gene_lists.asp) contained 23,644 probes with some annotation (Entrez gene ID or symbol) corresponding to 16,801 unique Entrez gene IDs. This initial annotation was updated using two sources: (1) the Agilent 06Sept2011 version of annotation file downloaded from Agilent eArray website (https://earray.chem.agilent.com/earray/) and (2) the annotation files downloaded from Rat Genome Database FTP website (http://ftp://rgd.mcw.edu/pub/data_release/) as of February 6, 2012. The Agilent probe ID was used as a common identifier whereby the annotation of 28,552 probes representing 18,157 unique genes was attained for a net gain of 4,908 additional annotated probes (1,356 unique Entrez gene IDs gained). The entire Entrez gene ID and their respective Symbols were verified from NCBI’s Gene webpage (http://www.ncbi.nlm.nih.gov/gene). This newly annotated list of probes is contained in Additional files 1 and 2: Table SA1 and Table SA2.

## Results

Male and female F344 rats aged 2 to 104 weeks (human equivalent of 1–3 months to 70–80 years) were sacrificed, and tissues collected at eight ages as previously described [19]. The gene expression in the kidney was measured using Agilent whole genome rat arrays. A combined statistical and fold-change cutoff value was used for the initial filtering criteria for both age and sex differences. Filtering for differential expression by age (ANOVA, FDR 5%, and fold change >1.5) resulted in 7,274 unique genes. Differential expression by sex (pairwise *t* test, FDR 5%, and fold change >1.5) resulted in 841 unique genes, showing sex difference at one or more ages, for a combined total of 7,447 unique DEGs by either age or sex in the kidney.

**Figure 1:**
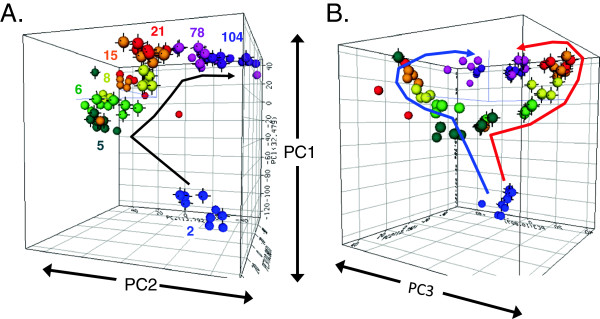
**Three-dimensional principal component analysis of differentially expressed genes.** The 7,447 differentially expressed features (FDR 5%; relative fold change >1.5) were used to assess the global view of each animal’s contribution to the life cycle expression profile. Each sphere represents the composite expression profile of one animal according to the top three principal components plotted in three-dimensional space (ArrayTrack). The spheres are colored by similar age group (*N* = 4 or 5). The spheres with black vertices indicate females, while those without indicate males. Animals generally cluster together according to respective age and sex in an age-sequential pattern. Figure 1A, B shows different views of the same plot: **(A)** highlights the age-related pattern, while **(B)** best displays the sex divergence in expression. The top three principal components accounted for 32.5%, 13.8%, and 10.8% of the total variance.

The 7,447 genes that met the filtering criteria were used for principal component analysis. Figure 1A,B illustrates the results using different views. The top three principal components (PCs) account for 32.5%, 13.8%, and 10.8% of the total variability, respectively, and are plotted in three dimensions to visualize the contribution of individual animals to the global expression profiles of the DEGs. Animals from the same age group and sex tend to cluster tightly with each other. Each age group appears in a pattern that is consistent with the sequential age in weeks (Figure 1A). PC1 accounts for variability due to age as the age groups separate along the axis parallel to PC1. Likewise, PC3 accounts for variability due to sex as the sex differences between animals of the same age group separate in space along the PC3 axis. PC2 appears to capture variability due to adult animal expression as 2- and 104-week groups differentiate from 8- to 21-week ones along the PC2 axis. Sex differences in the age groups begin to be most noticeable by 6 weeks of age (Figure 1B), are greatest at 15 and 21 weeks, and decrease at 78 and 104 weeks. Together, these data illustrate the relatively high reproducibility between biological replicates in a discrete and continuous linear pattern from young to old animals and among animals of the same sex. It also suggests sex differences in expression are most prominent during middle ages (8, 15, and 21 weeks) while still showing differences in old age (78 and 104 weeks).

**Figure 2:**
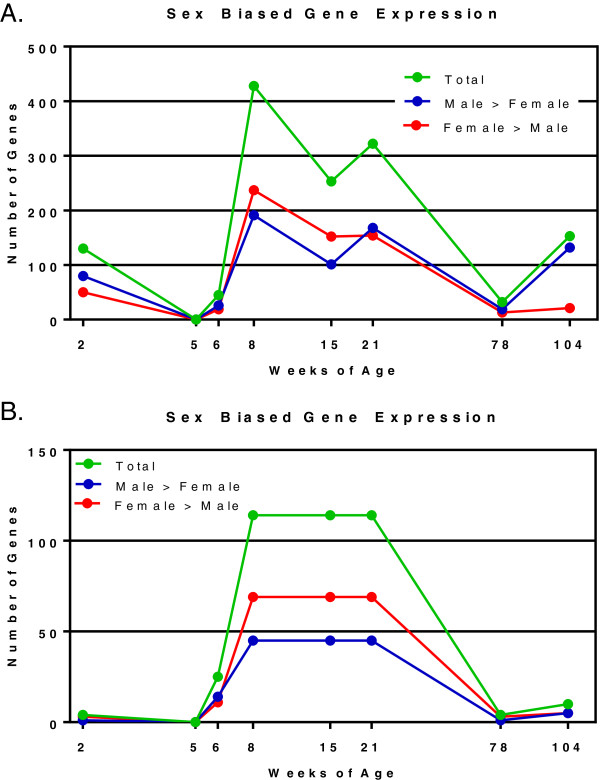
**Number of sex-biased genes per age group.** The number of unique, differentially expressed genes exhibiting sex-difference in expression (pairwise *t* test, FDR 5%, and 1.5 relative fold change between sexes) was calculated for each age group. The total number of sex-biased genes **(A)** is divided into female- and male-biased genes per age group. 114 sex-biased genes common to 8-, 15-, and 21-week animals **(B)** were plotted together in order to compare this list of robust sex differences accross all ages. Green, red, and blue lines represent numbers of total, female-biased, and male-biased genes, respectively, at each age group.

The gene expression differences between males and females during middle (8, 15, and 21 weeks) ages were further analyzed for sex differences using Venn analysis. The number of genes showing sex bias in expression (sex bias = pairwise *t* test FDR 5% and relative fold change >1.5 between sexes) was calculated at each age group. The number of male-biased (male expression levels higher than female) and female-biased (female expression levels higher than male) genes was plotted versus age (Figure 2A) to evaluate any patterns of unique sex bias during the rate life cycle. Three notable patterns were observed. First, 2-week-old animals exhibit 130 sex-biased genes prior to puberty. Secondly, those aging 8, 15, and 21 weeks exhibit the highest numbers of male-biased and female-biased genes, compared to other ages. Thirdly, sex-biased genes at 104 weeks exhibited substantially more male-biased genes (87% of all sex-biased genes) than female-biased genes.

**Figure 3:**
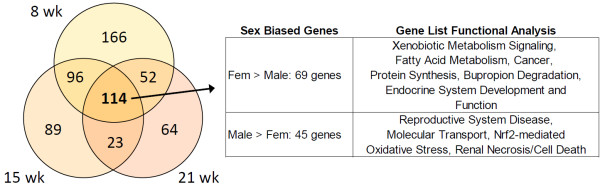
**Venn diagram analysis of sex-biased genes during middle age.** The intersection of genes exhibiting sex-biased expression at middle age groups (8, 15, and 21 weeks) was calculated using Venn analysis. The genes commonly sex-biased (i.e., Venn intersection) were divided into female- and male-specific gene lists, and functional analyses using Ingenuity Pathway Analysis was performed. Middle age groups (8, 15, and 21 weeks) showed relatively equal numbers of sex-biased genes. Sex-biased gene lists were analyzed for over-represented gene pathways or networks.

The evaluation of sex-biased genes at 2 weeks revealed 130 genes exhibiting male or female bias according to the criteria of 1.5-fold difference between males and females. However, for 80 of these genes (62%), males and females exhibit the same pattern of expression between 2 and 5 weeks. In addition, for these 80 genes, the age effect (fold change) between 2 and 5 weeks is greater than the sex effect (fold change) at 2 weeks. This means that age is having a greater influence on the expression at these ages than sex for these 80 genes. This suggests that the sex bias for more than half of the 130 two-week, sex-biased genes may be attributed to differences in timing or development between males and females rather than a true difference in gene expression. The 130 sex-biased genes at 2 weeks are listed in Additional file 3: Table SB. Because the middle ages (8, 15, and 21 weeks) showed large sex differences (as visualized in Figure 1B) and also comprise the most common ages for in-life rodent testing for preclinical toxicity studies, these three age groups were examined for genes showing consistently sex-biased expression. Venn analysis was used to identify 114 unique, sex-biased genes (Figure 3; see Additional file 4: Table SC for lists of the genes) common to all three age groups (i.e., Venn intersection). This set of 114 commonly sex-biased genes contains 69 female-biased genes and 45 male-biased genes. No genes in this set showed mixed or confounding patterns of sex-biased expression, meaning, all genes were consistently either male- or female-biased at all three age groups.

These 69 and 45 sex-biased genes were subsequently analyzed using Ingenuity Pathway Analysis for over-representation of specific functional pathways or networks. A consensus approach was used to review IPA’s Top Networks, Top Bio Functions, Top Canonical Pathways, and Top Tox Lists for repeated pathway or ontology terms for each list of genes analyzed (Figure 3). The 69 female-biased genes showed over-representation of xenobiotic metabolism, protein synthesis, endocrine system development and function, cancer, and fatty acid metabolism pathways. The 45 male-biased genes showed over-representation of reproductive system disease, molecular transport, Nrf2-mediated oxidative stress, and renal necrosis and cell death pathways. Thus, very different biology is reflected in these two sex-biased gene lists. The 114 sex-biased genes common to 8-, 15-, and 21-week animals were plotted together (Figure 2B) in order to compare this list of robust sex differences across all ages.

The examination of the sex-biased expression at 104 weeks revealed that 130 of the 152 sex-biased genes (87%) were male-biased. Functional analysis of the 130 male-biased genes revealed over-representation of immune response, inflammation response, immune cell trafficking, cell-mediated inflammatory response, and T cell receptor signaling pathways.

**Figure 4:**
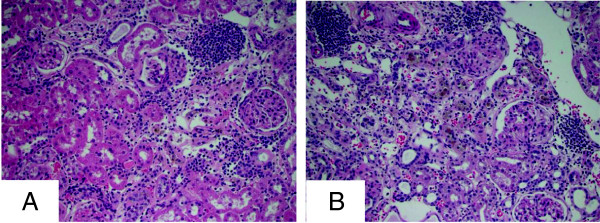
**Histopathological assessment of male-specific mononuclear cell infiltration.** Kidney sections (H&E stained) from male and female animals at 78 and 104 weeks were analyzed for presence and severity (scored 0–4 for absent to marked) of mononuclear cell infiltration, a common indicator of immune response and inflammation. Male animals were found to have higher severity scores on average than females at both 78 and 104 weeks. The figure shows kidneys of a female at 78 weeks **(A)** and a male at 78 weeks **(B)** exhibiting mild and moderate mononuclear cell infiltration, respectively.

The functional categories found for the male-biased gene expression pattern at 104 weeks of age suggested the strong activation of an immune reaction. To verify this at the cellular level, histopathological examination of kidney tissue sections was performed, and a 2- and 1.5-fold increase (M > F, *p* < 0.01) in average severity score for mononuclear cell infiltration was identified at 78 and 104 weeks, respectively (Figure 4). These histopathological findings confirmed the sex-specific (M > F) gene expression results that suggested increased immune cell response. Because such immune response may lead to the development of tissue fibrosis in older males, renal fibrosis was also evaluated by histological examination at 78 and 104 weeks. Fibrosis severity was scored from 0 to 4, with 0 indicating absent and 4 indicating marked. Males showed more fibrosis than females at these ages. Whereas female animals had an average fibrosis severity score of 0.5 (range 0–1) and 1.5 (range 1–3) at 78 and 104 weeks, respectively, males had an average fibrosis severity score of 2.1 (range 1–3) and 2.4 (range 1–4), respectively.

**Figure 5:**
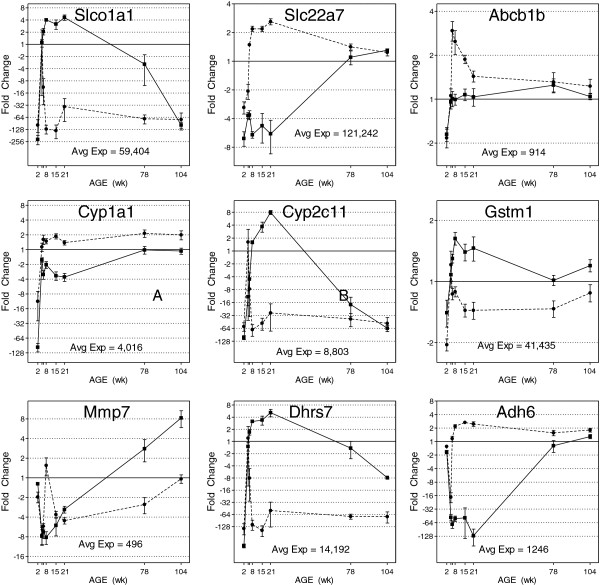
**Individual gene expression profiles exhibiting notable sex differences.** Genes encoding *Slco1a1*, Slc22a7, *Abcb1b*, *Cyp1a1*, *Cyp2c11*, *Gstm1*, *Mmp7*, *Dhrs7*, and *Adh6* exhibit notable sex differences in life cycle gene expression in the rat kidney. Relative fold changes (microarray data) are plotted; error bars represent SEM, *n* = 4 or 5. Females and males are indicated by dashed and solid lines, respectively. Averaged expression levels (normalized fluorescence intensities) across all age groups and in both sexes are reported for each gene and have been scaled to equal 1 in the plots.

Principal component analysis allows for the determination of those genes having the greatest influence on the variability in one principal component by rank ordering the loading values of that principal component. PC3 represents the variability within the dataset due to sex differences (Figure 1B). Analysis of the loadings values from PC3 revealed the top male- and female-biased genes influencing the dataset in the PCA. The female-biased genes having the largest influence on sex differences are alcohol dehydrogenase 6 (*Adh6*), *Col24a1*, and *Akr1b7*. The male-biased genes having the largest influence in sex differences are solute carrier organic anion transporter family member 1a1 (*Slco1a1*), dehydrogenase/reductase (SDR family) member 7 (*Dhrs7*), *Slc22a22*, cytochrome P450, family 2, subfamily C, polypeptide 11 (*Cyp2c11*), and *Mlc1*. Examples of individual gene expression profiles representing both the magnitude of sex differences observed and their potential relevance for kidney function are shown in Figure 5. The expression of the genes for Slco1a1, solute carrier family 22 member 7 (Slc22a7), and ATP-binding cassette, sub-family B (MDR/TAP), member 1B (Abcb1b), three crucial renal transporters involved in drug uptake and excretion, exemplifies the sex differences present, especially from 8 to 21 weeks. The maximum fold-change difference between the sexes ranges from 500 (male > female) for *Slco1a1* to 10 (female > male) for Slc22a7, and 3 (female > male) for Abcb1b. Cytochrome P450, family 1, subfamily A, polypeptide 1 (*Cyp1a1*), *Cyp2c11*, and glutathione S-transferase theta 1 (*Gstm1*) encode key phase I and II drug metabolism proteins, with fold-change expression differences between the sexes of approximately 6 (female > male), 250 (male > female), and 2 (male > female), respectively. Matrix metallopeptidase 7 (*Mmp7*), an extracellular matrix maintenance enzyme implicated in fibrosis, exhibits male-specific up-regulation in the late age groups (eightfold). *Dhrs7* and *Adh6*, coding for two dehydrogenases, represent the most influential sex-biased genes according to PCA loading value analysis, with *Dhrs7* showing a 500-fold male-biased expression and *Adh6* showing a 250-fold female-biased expression. These examples illustrate the magnitude and timing of the sex-biased expression of genes with toxicological and pharmacological relevance during the life cycle.

**Figure 6:**
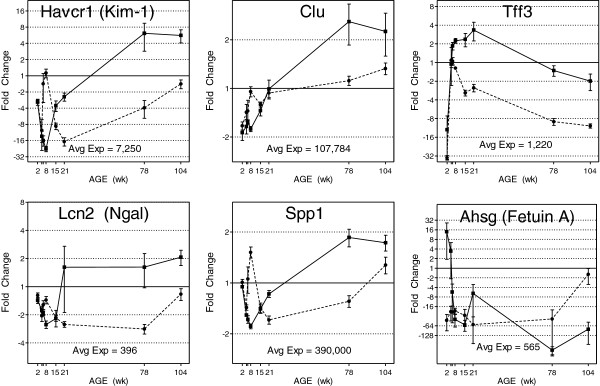
**Differential life cycle expression of genes encoding qualified and proposed kidney biomarkers.** Genes encoding qualified renal biomarkers Kim-1, Clu, Tff3, Lcn2, Spp1, and proposed renal biomarker Fetuin A show sex differences in expression from 2- to 600-fold in the kidney. Relative fold changes (microarray data) are plotted; error bars represent SEM, *n* = 4 or 5. Females and males are indicated by dashed and solid lines, respectively. Averaged expression levels (normalized fluorescence intensities) across all age groups and in both sexes are reported for each gene and have been scaled to equal 1 in the plots.

Several gene products have been proposed recently for use as kidney safety biomarkers [17,21], six of which have been qualified by the FDA for use in preclinical monitoring of disease- and drug-induced nephrotoxicity (Table 1). The expression levels of five of the six genes encoding qualified biomarkers exhibit sex differences of twofold or greater and include kidney injury molecule 1 (*Kim-1*), clusterin (*Clu*), trefoil factor 3c (*Tff3*), osteopontin (*Spp1*), and lipocalin 2 (*Lcn2*) (Figure 6). Notably, *Kim-1* exhibits about 23-fold difference at 8 weeks, the most common age for in-life toxicity evaluations. Fetuin A, a proposed renal biomarker, exhibits sex differences in gene expression of over 600-fold at 2 weeks (Table 1). These gene expression differences suggest notable sex-related changes in protein expression which may impact their use and utility. Taken together, further evaluation of the role of sex differences in renal biomarker discovery, qualification, and use is warranted.

**Table 1:** Current qualified and proposed kidney injury biomarkers

**Kidney biomarker**^**a**^	**Qualified**	**Kidney injury location**	**Sex bias**	**Maximum fold-change sex difference**	**Age of largest sex difference (weeks)**
Kim-1 (Havcr1)	Yes	Prox Tub	F > M	23.5	8
TFF3 (Tff3)	Yes	Prox Tub	M > F	9.7	21
NGAL (Lcn2)	Yes	Prox, Dist Tub	M > F	3.7	78
Osteopontin (Spp1)	Yes	Prox, Dist Tub, Lp Hnle	F > M	2.7	8
Clusterin (Clu)	Yes	Prox, Dist Tub	M > F	2	104
GST-α (Gsta2)	Yes	Prox Tub	No sex difference	-	-
Fetuin-A (Ahsg)	No	Prox Tub	M > F	600	2
HGF (Hgf)	No	Prox Tub	F > M	2.4	2
GST-μ (Gstm1)	No	Dist Tub	M > F	2.1	21
Interleukin-18 (*Il-18*)	No	Prox Tub	M > F	1.5	104
GST-π (Gstp1)	No	Dist Tub	No sex difference	-	-
NHE3 (Slc9a3r2)	No	Prox Tub, Lp Hnle	No sex difference	-	-
Liver-type FABP (Fabp1)	No	Dist Tub	No sex difference	-	-
CYR-61 (Cyr-61)	No	Prox Tub	No sex difference	-	-
Heart-type FABP (Fabp3)	No	Dist Tub	No sex difference	-	-
NAG (Hexb)	No	Prox Tub	No sex difference	-	-
Netrin-1 (Ntn1)	No	Prox Tub	No sex difference	-	-

*Prox Tub* proximal tubules, *Dist Tub* distal tubules, *Lp Hnle* loop of Henle.

^a^Biomarkers are referenced by their most common referenced human gene name followed by the official rat gene symbol in parentheses.

## Discussion

Whole genome microarray gene expression data provide a broad view of molecular activities in the rat kidney. With an understanding that mRNA expression levels do not necessarily correlate with protein expression levels, the data are interpreted in view of both relative fold-change differences in expression as well as the mean normalized intensity for each gene as a starting point for assessing biological differences. Genes with higher mean expression will provide more robust and reliable fold-change estimates than weakly expressed transcripts. It is understood that stressor-induced responses in gene expression may exhibit different characteristics than basal level gene expression with respect to the degree of sex differences observed. Furthermore, genes showing similar basal expression between sexes may exhibit substantial sex differences in response to stressors. Three-dimensional principal component analysis provides a global view of the relative impact of sex differences on the variability of gene expression during the rat life cycle. Even though PC3 accounts for less than 11% of the variability of the differentially expressed genes, sex differences are clearly evident between 8 and 21 weeks of age (Figure 1B). The rank ordering of the loading values for the principal components provides a starting point in assessing which genes are most responsible for the differences between sexes and organizes the genes into male- and female-biased gene lists. For example, *Adh6* exhibits the largest influence on sex differences for female-biased expression with an expression level of more than 250-fold greater in females than in males. *Adh6* metabolizes a variety of substrates including ethanol, retinol, hydroxysteroids, and lipid peroxidation products. *Adh6*’s dramatic sex-specific expression in the kidney has not been previously reported, and its physiological relevance is not clear.

The number of genes exhibiting sex-biased expression varies across the eight ages with the exception of 5- and 78-week-old rats, when there is a substantial decrease (Figure 2A). Two-week animals exhibit a considerable number of sex-biased genes, given the general lack of sex-hormone control. The notable drop in sex-biased genes at 5 weeks of age may be a function of decreased sex-specific signaling during the perinatal stage, shortly after 2 weeks, and just prior to pubertal surges in steroid hormones at 6 weeks which produce sexually mature rats by 8 weeks of age [22]. This is consistent with the rapid increase in sex-specific genes observed at 6 and 8 weeks of age. Prior to 104 weeks of age, there are approximately equal numbers of male-biased and female-biased genes at each age. The increase in the number of genes whose expression is male-biased and the decrease in the number of genes whose expression is female-biased at 104 weeks appear to be explained by male-specific renal fibrosis, inflammation, and immune cell infiltration [14]. Immune response genes (*Tgfb1*, *Il-18*, *Il-1b*, *Il-22*, *Il-9r*, and *Il-2rg*;) show male-biased, old-age-dependent increases in expression at 78 and 104 weeks of age. Histopathological evaluation of the 78- and 104-week kidney sections confirmed the male-biased presence of infiltrating mononuclear cells, indicative of immune cell activity. The higher level expression of these genes in males compared to females may be responsible for the finding that females show protection against age-related fibrosis and inflammation [4].

Venn analysis, using the intersection of age-specific sex differences, follows a consensus approach to minimize false positives of genes truly exhibiting sex differences at multiple consecutive age groups. However, this approach does remove genes that would be *bona fide* sex-biased only transiently at single age groups. Of the 69 common female-biased genes from the 8- to 21-week age groups, the top represented canonical pathway was the bupropion degradation pathway that contains key enzymes cytochrome P450 4B1 and 4F8 which, respectively, exhibit greater than 8- and 16-fold increased expression in female versus male rats. Phase 1 metabolizing enzyme Cytochrome P450 1A1 also showed female-biased expression (6.7-fold F > M) at 8–21 weeks of age, suggesting potential sex differences in metabolism of polycyclic aromatic hydrocarbons. Also represented between 8 and 21 weeks were three UDP-glucuronosyltransferase family members (*Ugt2b17*, *Ugt2b4*, and *Ugt2b7*) which are phase 2 conjugating enzymes with roles in metabolizing steroid hormones (estrogen metabolites) and drugs or xenobiotics. Notably, *Ugt2b7* is responsible for the metabolism of morphine into an inactive form. The expression of aldo-keto reductase family 1, member C1, which catalyzes the conversion of progesterone into its inactive form 20-alpha-hydroxy-progesteronem, was also found to be female-biased. Bradykinin/kallidin pathway component *Kng2* (kininogen 2, kallidin, des-Arg10-kallidin, bradykinin) integrates with the Ras (rat sarcoma gene family) pathway in modulating normal renal function and showed 9-fold higher expression in females than males. Putative sex hormone regulation of kallikrein-related gene expression may be linked to the human pancreatic/renal kallikrein gene having potential estrogen and progesterone receptor binding sites and higher urinary kallikrein excretion in women; renal kallikrein and kallikrein mRNA levels are lowered after ovariectomy in rats [23]. The 45 common, male-biased genes were most represented by reproductive system disease-related genes (*Apoh, Esr1, F2, Prlr, Abcg2, Kifc1, Rasl12, Gstm5*), with estrogen receptor alpha being expressed >3-fold higher in males than females between 8 and 21 weeks of age. Steroid hormone metabolism enzymes 3-beta-hydroxysteroid dehydrogenase 1 and 2 and estradiol-17-beta dehydrogenase 1 all exhibited male-biased expression, consistent with their roles in maintaining steroid levels in a sex-specific manner.

Of the 74 cytochrome P450 enzymes present on the Agilent rat microarray, 24 of them (32%) exhibit some level of sex bias in expression, suggesting important differences that may underlie drug-induced susceptibilities. For instance, *Cyp2c11* shows a >250-fold higher expression in males compared to females, suggesting sex-dependent susceptibility to certain drug or xenobiotic substrates of Cyp2c11. For example, limonene is a substrate for Cyp2c11 [24] and has been shown to be a kidney carcinogen in the male rat, but not in the female rat [25]. Cyp2c11 is involved in the testosterone metabolism pathway (formation of 2a- and 16a-hydroxytestosterone) and is inhibited by Bisphenol A (BPA) in rat liver [26]. Despite the relatively high dose of BPA used to elicit this effect *in vitro*, the sexually dimorphic expression of Cyp2c11 may be important in mediating the potential endocrine-disrupting health effects of BPA.

The effects of xenobiotic and drug exposures on the handling of renal and extra-renal metabolites and their transport contribute to the major reasons for kidney toxicity. Tissue or cellular levels of the molecular transporters that mediate the selective filtering of these molecules are linked to the kidney’s role in retaining or excreting these metabolites. A recent review [27] lists six (five uptake and one efflux) important kidney transporters which have been reported to exhibit sex differences in expression in the rat kidney. Four of these six exhibited matched, sex-biased expression at the mRNA level in the current dataset including *Slco1a1*(OATP1A1), Slc22a7(OAT2), Slc22a2(OCT2), and Abcg2(BCRP). The two proximal tubule apical uptake transporters Slco1a1 and Slc22a7 [27] showed opposite sex-biased gene expression. Slco1a1 exhibited >500-fold, male-biased expression between 6 and 21 weeks, while Slc22a7 shows up to 17-fold, female-biased expression at the same ages (Figure 5). These two transporters are responsible for the handling of a significant proportion of prescribed drugs [1]. However, an additional eight multidrug resistance-related transporters were also shown to exhibit sex biases in expression, including Abcb1b (Figure 5), Abcc8, Slc22a3, Slc21a4, Slco1a5, Slco1a6, and Slco1c1. Slco1a5 and Slco1a6 are rodent-specific organic anion transporters that show a fourfold or twofold male-biased expression difference, respectively, between 8 and 21 weeks of age, which may limit or complicate the translation to clinical relevance. However, the extrapolation of the sex-biased expression of Slc21a4 to humans may be more promising. Slc21a4(OAT-K1) is a kidney-specific organic anion transporter that facilitates the basolateral uptake and clearance of methotrexate, a drug prescribed for autoimmune diseases as well as cancer chemotherapy. Our data show that Slc21a4 exhibits male-biased expression (around twofold) between 8 and 21 weeks of age. This sex-biased expression pattern may provide explanation for clinical observations of sex-dependent pharmacokinetics of methotrexate in CNS-lymphoma patients [28]. In the clinic, females show significantly decreased clearance of methotrexate compared to males, which is consistent with lower expression of this transporter in female rats. The functional impact of mRNA expression differences between males and females requires further investigation for genes encoding these important transporters. However, our data present several novel genes exhibiting sex-biased expression in the kidney for further evaluation of functional differences between males and females.

**Figure 7:**
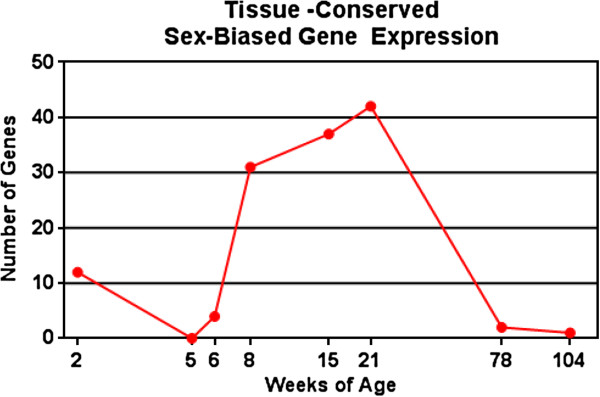
**Comparison of sex-biased genes between kidney and liver.** The number of sex-biased genes was calculated at each age group for the kidney and compared to previously published data in the liver [19]. The number of sex-biased genes common between kidney and liver is displayed for each age group. A list of the 153 sex-biased genes common to both tissues is available in Additional file 5: Table SD.

Sex differences in rat life cycle liver gene expression have been previously published [19]. Identifying sex differences between liver and kidney is of interest to evaluate sex bias in drug metabolism and excretion. A tissue comparison of sex-biased gene expression was performed to identify such tissue-conserved sex differences. The previously published liver data was re-analyzed using the same criteria (pairwise *t* test, FDR 5%, and 1.5-fold change) for differential expression as the current kidney data. This analysis resulted in a total of 1,125 liver genes showing sex-biased expression at one or more ages compared to the 841 sex-biased kidney genes. There were 153 genes in common between the two tissues (Additional file 5: Table SD). To evaluate if sex bias in gene expression was present at the same ages in each tissue, a comparison was performed at each age (Figure 7). As 8, 15, and 21 weeks exhibited the highest numbers of tissue-conserved, sex-biased genes, a comparison of the three ages was performed revealing ten genes in common between the three ages. These ten genes are: *Adh6*, *Akr1b7*, *Dhrs7*, *LOC690226*, *Odf3b*, *Prlr*, *Scml4*, *Tmed3*, *Trim24*, and *Ust5r*. It is interesting to note that *LOC690226* is currently annotated as “similar to *Dhrs7*” but is located at a unique locus on the same chromosome (Chromosome 6) as *Dhrs7*. All genes exhibited the same sex bias between liver and kidney except Prlr. In the kidney, Prlr shows male-biased expression, whereas in the liver, Prlr shows female-biased expression. It is unclear what influence these tissue-conserved, sex-biased gene expression differences may have on sex-specific susceptibility to adverse health effects or diseases. One previously published report [29] compared sex-biased expression of drug metabolism-related genes between liver and kidney in rats of 8 weeks of age. Only six genes (*Cyp2c6*, *Cyp2c11*, *Cyp2c12*, *Ugt1a8*, *Ugt1a9*, and *Ahr*) were reported as having conserved sex-biased expression between liver and kidney. Of these six genes, only *Cyp2c11* was in common with the tissue-conserved, sex-biased genes identified in the rat life cycle studies. *Cyp2c11* exhibits male-biased expression during middle ages in both liver and kidney which has been well characterized [30].

The qualification of renal urinary proteins as preclinical biomarkers of acute kidney injury has proceeded mostly in the absence of female data [17]. Thus, the analysis of potential sex-specific differences in biomarker behavior or performance will benefit their use and application. As previously reported, the age-specific nephrotoxicity observed after cisplatin and gentamicin treatment in male animals [31] correlated well with Havcr1(KIM-1) expression levels in the kidney [19]; increasing levels of nephrotoxicity correlated with increasing mRNA expression of KIM-1, depending on the animal age. The performance of KIM-1 as a biomarker of such nephrotoxicity for female animals might require further investigation because mRNA expression of KIM-1 in female animals at 8 weeks shows >23-fold sex difference (F > M) (Figure 6; Table 1). The finding of a large sex bias in the expression of the *KIM-1* gene (23-fold higher in females than in males) cautions against direct extrapolation of the male preclinical biomarker qualification data to females. The genes coding for the qualified renal injury biomarkers Tff3 and Spp1 also exhibited sex-biased expression (9.7- and 2.7-fold sex bias, respectively; Figure 6, Table 1) at 8 to 21 weeks of age, the ages most commonly used for in-life toxicology studies. These data provide comprehensive life cycle coverage of sex-specific mRNA expression levels not previously reported, which should provide sufficient evidence for the investigation of further sex-specific functional differences in kidney urinary protein biomarker performance as well as the enzymes and molecular transporters implicated in acute kidney injury and chronic kidney disease.

## Conclusions

These data provide evidence of sex-related differences in the expression of genes in the kidney of rats over the life cycle, from 2 weeks of age to 2 years of age. Many of the genes showing sex-biased gene expression may impact gene networks crucial to biological pathways, such as xenobiotic metabolism (*Cyp* enzymes and drug transporters), inflammation, and fibrosis that may underlie sex-related susceptibilities. Gene-based biomarkers are being evaluated and qualified for preclinical testing because early diagnosis of drug-induced kidney injury is a key consideration for pharmaceutical safety and decision-making. Currently, proteins encoded by six genes (*KIM-1*, *Clu*, *Tff3*, *Spp1*, *Lcn2*, and *Gsta2*) have been qualified by the FDA for use as urinary biomarkers of kidney injury, and additional markers (Table 1) continue to be evaluated. We describe dramatic sex differences in the expression of genes encoding these renal urinary biomarkers during the life cycle, and consideration of these data should provide a stronger basis for evaluating all renal biomarkers being proposed. Together, these sex-related changes in kidney gene expression during the rat life cycle expand current understanding of kidney function and inform the use of current and proposed safety biomarkers.

## Abbreviations

Cyp: Cytochrome P450; DEGs: Differentially expressed genes; F344: Fischer 344; FDA: Food and drug administration; IPA: Ingenuity pathway analysis; NCBI: National center for biotechnology information; PCA: Principal component analysis; PC: Principal component; RIN: RNA integrity number; URR: Universal reference RNA.

## Competing interests

The authors declare that they have no competing interests.

## Authors’ contributions

JCK carried out the microarray experiments, data processing, and pathway analysis, and drafted the manuscript. VGD participated in the study’s conception and in the design and coordination of the in-life study. VV performed annotation update for gene expression data. CLM participated in the in-life study and RNA isolation. JCF conceived the study, participated in its design and coordination, and helped draft the manuscript. All authors read and approved the final manuscript.

## Supplementary Material

Additional file 1: Table SA1 Complete gene expression dataset Part1. Complete dataset with annotations, fold changes, and statistical values. Click here for file

Additional file 2: Table SA2 Complete gene expression dataset Part2. Complete dataset with annotations, fold changes, and statistical values Click here for file

Additional file 3: Table SB 130 sex-biased genes at 2 weeks. List of sex-biased genes at 2 weeks. Click here for file

Additional file 4: Table SC Venn analysis of 8, 15, and 21 weeks. Click here for file

Additional file 5: Table SD 153 sex-biased genes common to kidney and liver. Click here for file
